# Diabetes mellitus in North West Ethiopia: a community based study

**DOI:** 10.1186/1471-2458-14-97

**Published:** 2014-01-30

**Authors:** Solomon Mekonnen Abebe, Yemane Berhane, Alemayehu Worku, Abebayehu Assefa

**Affiliations:** 1 Department of Physiotherapy, College of Medicine and Health Sciences, University of Gondar, Gondar, Ethiopia; 2 Addis Continental Institute of Public Health, Addis Ababa, Ethiopia; 3 School of Public Health, Addis Ababa University, Addis Ababa, Ethiopia; 4 World Health Organization, Country Office- Ethiopia, Addis Ababa, Ethiopia

## Abstract

**Background:**

Diabetes mellitus is recognized as one of the emerging public health problems in developing countries. However, its magnitude has not been studied at community levels, making the provision of appropriate services difficult in such countries. Hence, this study aimed to compare the magnitude and associated risks of diabetes mellitus among urban and rural adults in northwest Ethiopia.

**Methods:**

A cross-sectional population based survey was performed using the WHO STEPwise method on adults aged 35 years and above. A multistage cluster random sampling strategy was used to select study participants from urban and rural locations. Fasting blood glucose levels were determined using peripheral blood samples by finger puncture. Prevalence was computed with a 95% confidence interval for each residential area. Selected risk factors were assessed using logistic regression.

**Results:**

The prevalence of diabetes mellitus among adults aged 35 years and above was 5.1% [95% CI: 3.8, 6.4] for urban and 2.1% [95% CI: 1.2, 2.9] for rural dwellers. The majority (69%) of the identified diabetic cases were not diagnosed prior to the survey. The highest proportion (82.6%) of the undiagnosed cases was noted among the rural population and 63% among the urban population. Family history of diabetes (AOR = 5.05; 2.43, 10.51), older age (AOR = 4.86; 1.99, 11.9) and physical inactivity (AOR = 1.92; 1.06, 3.45) were significantly associated with diabetes mellitus among the urban population. Alcohol consumption (AOR = 0 .24, 0 .06, 0.99) was inversely associated with diabetes mellitus in rural areas.

**Conclusion:**

The prevalence of diabetes mellitus is considerably high among the urban compared to the rural population. Diabetes is largely undiagnosed and untreated, especially in rural settings. Appropriate actions need to be taken to provide access to early diagnosis and treatment in order to reduce associated complications.

## Background

Diabetes Mellitus (DM) is a metabolic disorder characterized by chronic hyperglycemia [1]. The global burden of diabetes has increased twelve fold between 1985 and 2011[2,3]. The International Diabetes Federation (IDF) suggests that the number of adults living with diabetes worldwide will further expand by 50.7% by 2030 [2]. Evidence shows that DM is claiming the lives of more than 4 million people worldwide annually and developing countries account for a substantially high proportion [4].

According to the 2011 report of the International Diabetes Federation (IDF), the number of adults living with diabetes in Ethiopia was 3.5% [3]. A study done on urban Commercial Bank employees in Ethiopia showed a 6.5% prevalence of DM [5] which indicated the significance of lifestyle for DM etiology. However, population-based estimations, especially for rural settings are lacking.

Physical activity and over-weight have not received much importance in the daily life of the urban population in Africa due to the prevailing inadequate road infrastructure necessitating long distance walking on a daily basis. However, with the progressive improvement in roads, the adoption of western lifestyle, and the rising number of ageing population, people in urban areas are more prone to developing diabetes [6]. So far, most of the diabetes mellitus studies in Ethiopia were institution-based and urban focused with no studies of rural dwellers. Hence, population-based epidemiological information from both urban and rural populations is essential to understand the whole picture of DM in Ethiopia [5,7-9] to help expand service availability and appropriate clinical intervention. Therefore, assessing and comparing the prevalence of DM in the urban and rural areas of Ethiopia is imperative in order to inform, in particular, the governmental and clinical decision makers [10]. The objective of the study was thus to assess the prevalence and associated risk factors of DM among rural and urban dwellers in northwest, Ethiopia.

## Methods

### Study population

This study was conducted in two residential settings in the town of Gondar and in a largely rural district of Dabat. Gondar town has an estimated population of 210,000 and is one of the largest towns of the country [11]. The rural district of Dabat DHSS has an estimated population of 44,723 that is largely living on subsistance farming [12].

The study utilized a comparative cross-sectional community-based design. All permanent residents in the study area who were 35 years and older were eligible to participate in the study.

The sample size for the study was determined by assuming the prevalence of urban DM to be 2.0% and that of rural DM to be 0.5% [13]. A power of 80% was used to detect the difference indicated between the urban and rural populations, with a 95% confidence level and 10% of non-response rate. Accordingly, the calculated sample size was 1100 for urban and 1100 for rural settings [14]. A multistage cluster random sampling strategy was used to select study participants from urban and rural locations. Initially, clusters targeting the smallest administrative units in Ethiopia called ‘Kebele’ were selected using simple random sampling after obtaining the list from the district administration. Then, households were selected within each cluster using the systematic random sampling technique. Finally, one eligible adult was selected from each household using simple random sampling.

### Data collection

Data were collected by interviewing eligible subjects using a pretested and structured questionnaire. The questionnaire, which was in the local language, included questions that assessed diabetic risk factors. Fasting blood glucose was measured as per the WHO recommendations [15]. Peripheral blood samples by finger puncture were collected early in the morning before participants took their breakfast. The WHO and IDA criteria were used to classify fasting blood glucose levels [16,17]. Anthropometric measurements were taken using standardized techniques and calibrated equipment. Subjects were weighed to the nearest 0.1 kg in light indoor clothing and bare feet or with stockings. Height was measured using a stadiometer; participants stood in erect posture without shoes, and the results were recorded to the nearest 0.5 cm. Measures were taken two times, and the average was considered in the analysis. Body mass index (BMI) was calculated as the ratio of weight in kilograms to the square of height in meters. Waist girth was measured by placing a plastic tape to the nearest 0.5 cm horizontally, midway between the 12th rib and iliac crest on the mid-axillary line. Hip circumference was measured around the widest portion of the buttocks, with the tape parallel to the floor [18].

House-to-house data collection was performed by trained field workers. The field study team was composed of enumerators, laboratory technicians, nurses, and supervisors. All were trained by the principal investigator for three days on the study procedures. To ensure the quality of the interview and the acquisition of quality data, random checks were carried out by the principal investigator.

### Data analysis

Double data entry procedures were done using the EPI Info statistical software. The prevalence estimations for urban and rural subjects were made separately by sex. The prevalence estimation was made along with a 95% confidence interval (CI). The results were considered statistically significant at P ≤ 0.05. Logistic regression was applied to identify risk factors for urban and rural participants separately. The independent variables were selected based on prior evidence in the literature and their effect in current analysis. Independent variables with a p-value of 0.20 and less during the bivariate test were then included in the multivariable logistic regression model.

Statistical analysis was performed using the STATA version 11 software. Fasting plasma glucose (FPG) > 126 mg/dl was used to make the diagnosis of DM, which was confirmed by repeating the test on another day [15].Waist circumference (WC) was categorized as low risk if it was 93.9 cm or less for men, and 79.9 cm or less for women; high risk if it was 94 cm or more for men, and 80 cm or more for women [19].

Study participants were recruited voluntarily after obtaining full information about the study and signing a written consent agreement. They were informed of their rights to withdraw from the study at any stage. Individuals identified as cases of DM were referred to the nearby clinic for further treatment and follow-up. The protocol and written consent was approved by the Institutional Review Board of the University of Gondar.

## Results

A total of 1100 study participants were initially enrolled from each urban and rural setting, but some refused to give a blood sample, and were excluded. The respondent rate was 97.3%. Thus, 1050 study subjects from urban and 1091 rural areas were included in the analysis. The Mean age (±SD) of the urban population was 49.9 (±12) years and that of the rural population was 46.6 (±3) years. The socio-demographic and life style characteristics of the study participants were stratified by sex and residence (Table 1).

**Table 1:** Socio-demographic characteristics of the study population by residence and sex for adults age 35 and above years, North West Ethiopia, 2012

**Variable**	**Study participant**				**Diabetic case n (%)**
	**Urban**		**Rural**		
	**Male n (%)**	**Female n (%)**	**Male n (%)**	**Female n (%)**	
**Age group (years)**					
35-44	142 (40.5)	265 (37.9)	280 (44.4)	215 (46.6)	20 (2.22)
45-54	99 (28.2)	201 (28.8)	159 (25.2)	107 (23.2)	16 (2.83)
55-64	39 (11.1)	139 (19.9)	103 (16.4)	84 (18.2)	21 (5.75)
≥ 65	71 (20.2)	94 (13.5)	88 (13.9)	55 (11.9)	20 (6.49)
**Religion**					
Orthodox	311 (89.9)	654 (94.5)	624 (99.1)	455 (98.7)	73 (3.57)
Muslim	32 (9.2)	36 (5.2)	6 (0.9)	6 (1.3)	4 (5.00)
Others	3 (0.9)	2 (0.3)	0 (0.00)	0 (0.00)	0 (0.00)
**Level of education**					
Non formal school	137 (39)	438 (62.7)	544 (86.4)	437 (62.7)	57 (3.66)
Grade 1-6	23 (6.6)	50 (7.2)	75 (11.9)	20 (4.3)	8 (4.76)
Grade 7-12	102 (29.1)	138 (19.7)	9 (1.4)	3 (0.65)	6 (2.38)
Diploma and above	68 (19.4)	20 (2.9)	1 (0.2)	0 (0.00)	4 (5.80)
Refused	21 (5.9)	53 (7.6)	1 (0.2)	1 (0.22)	4 (3.23)
**Marital status**					
Never married	36 (10.3)	49 (7.02)	5 (0.8)	0 (0.00)	4 (4.44)
Currently married	269 (77.1)	412 (59)	608 (96.5)	278 (60.3)	52 (3.32)
Separated	18 (5.2)	47 (6.7)	1 (0.2)	5 (1.1)	6 (8.45)
Divorced	9 (2.6)	60 (8.6)	6 (0.95)	34 (7.4)	6 (5.5)
Widowed	10 (1.6)	129 (18.5)	10 (1.6)	144 (31.2)	9 (3.01)
**BMI kg/m2**					
≤ 18	26 (7.4)	80 (11.5)	183 (29.1)	155 (33.7)	8 (1.8)
18 - 24	257 (73.2)	460 (66)	435 (69.1)	299 (65)	49 (3.38)
25 and above	68 (19.4)	157 (22.6)	12 (1.9)	6 (1.3)	19 (7.82)
**Family history**					
Yes	39 (5.6)	35 (10.03)	4 (0.9)	0 (0.00)	14 (17.95)
No	658 (94.4)	314 (89.97)	456 (99.1)	630 (100)	63 (3.06)
**Smoking**					
Yes	18 (5.1)	0 (0.00)	5 (0.8)	1 (0.22)	0 (0.00)
No	333 (94.9)	697 (100)	625 (99.2)	460 (99.8)	77 (3.64)
**Alcoholic drink within the past 30 day**					
Yes	140 (39.9)	104 (14.9)	619 (98.2)	441 (95.7)	27 (2.07)
No	211 (60.1)	595 (85.1)	11 (1.75)	20 (4.3)	50 (5.97)
**Frequency of eating fruit in a typical week**					
Not eating	56 (16.1)	134 (19.4)	625 (99.4)	455 (98.7)	31 (2.44)
1-3 times	279 (79.9)	530 (76.6)	4 (0.6)	4 (0.9)	41(5.02)
4-7 times	14 (4.01)	28 (4.1)	0 (0.00)	2 (0.43)	3 (6.82)
**Frequency of eating vegetables in a typical week**					
Not eating	19 (5.5)	43 (6.2)	628 (99.7)	456 (98.9)	25 (2.18)
1-3 times	265 (77.3)	492 (70.7)	2 (0.32)	4 (0.9)	37 (4.82)
4-7 times	60 (17.2)	161 (23.1)	0 (0.00)	1 (0.22)	15 (6.76)

About 8.8% of the study population was overweight, and 2.5% was obese. Over-weight and obesity were higher among the urban community (21.5%) than the rural ones (1.66%). The proportion was higher (22.6%) among females than males (19.4%) in the urban community. The proportion was low (1.9%) among males and (1.3%) among females in the rural community. The proportion with abdominal obesity was high (37.7%) among urban subjects than the rural subjects (25%). The proportion was higher among women (49.1%) than men (10.2%). The proportion of alcohol drinking during the last 30 days in the study population was higher among rural males (98.2%) and females (95.7%) than the urban males (39.9%) and females (14.9%). The proportion of cigarette smoking among the study group was very low (1.1%). In a typical week, 3.6% ate fruit; the highest proportion (7.08%) was noted among urban residents and a very low proportion (0.37%) among rural dwellers (Table 1).

### Prevalence of diabetes mellitus

**Figure 1:**
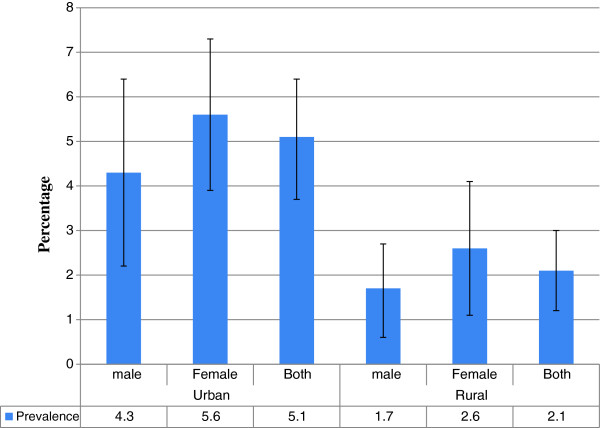
**Prevalence of diabetes mellitus by residence and sex with 95% CI error bar among adults age 35 and above years, North West Ethiopia, 2012**.

**Figure 2:**
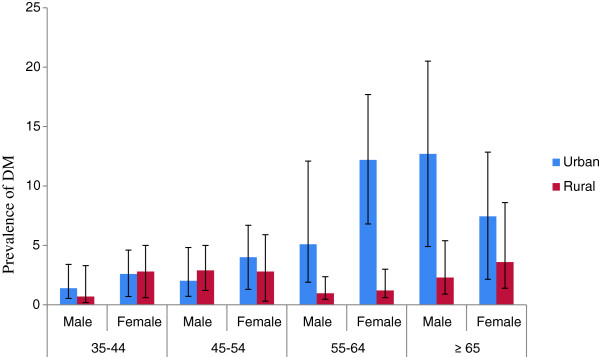
Prevalence of diabetes mellitus by Socio-demographic characteristics and residence of the study population among adults age 35 and above years, North West Ethiopia, 2012.

The prevalence of diabetes mellitus was 2.1% [95% CI 1.25 – 2.96] in rural areas and 5.1% [95% CI: 3.80 – 6.48] in urban areas (Figure 1). In rural areas, the prevalence was 1.7% among men and 2.6% among women. In urban areas, the prevalence was 4.3% among men and 5.6% among women. There was no statistically significant difference between males and females in rural and urban areas (Figures 1 & 2).

**Figure 3:**
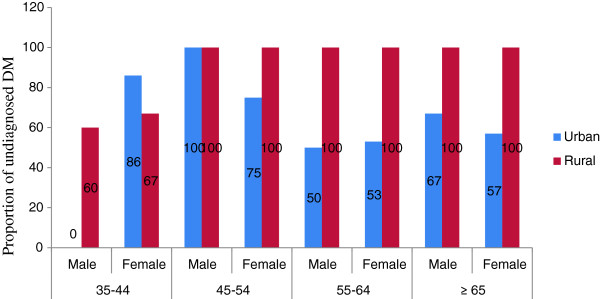
Previously undiagnosed diabetes mellitus by age, sex of the study population and residence among adults age 35 and above years, North West Ethiopia, 2012.

The proportion of previously undiagnosed DM was 53 (69%); which was 82.6% (19 of 23) in rural areas and 63% (34 of 54) in urban areas. In the rural areas, the proportion of previously undiagnosed DM tended to rise as the age of the population increased (Figure 3).

The multivariate logistic regression analysis of factors associated with diabetes mellitus among the male and female sex is presented in Table 2. The logistic regression analysis done separately for urban and rural participants revealed associated risk factors. In the urban population, DM was significantly associated with family history of DM (AOR = 5.05; 2.43, 10.51), older age (AOR = 4.86; 1.99, 11.9), and physical inactivity (AOR = 1.92; 1.06, 3.45). Alcohol consumption (AOR = 4.16; 1.02, 17.04) was inversely associated with diabetes mellitus in the rural population. Moderate alcohol drinking for the last 30 days was protective (AOR = 0 .24, 0 .06, 0.99) for DM (Table 3). After stratifying the data according to sex controlling for residence, age 65 and above was significantly associated with diabetes mellitus among males (AOR = 3.55; 1.30, 9.72). Family history of DM (AOR = 7.1; 3.07, 16.25), physical inactivity (AOR = 2.1; 1.09, 4.02) and age 55–64 (AOR = 2.2; 1.07, 4.4), were significantly associated with diabetes mellitus among females (Tables 2 and 3).

**Table 2:** Multivariate analysis of factors associated with diabetes mellitus among male and female sex of Gondar, North West Ethiopia (2012)

	**Adjusted OR [95% CI]**	
**Variable**	**Male**	**Female**
**Age in year**		
35-44	1.00	1.00
45-54	1.00 [0.31,3.24]	1.28 [0.54,3.03]
55-64	1.33 [0.34,5.25]	2.72 [1.24,5.97]
≥ 65	3.55 [1.30,9.72]	2.29 [0.92,5.70]
**Residence**		
Urban	1.00	1.00
Rural	0.84 [0.25,2.77]	1.57 [0.48,5.09]
**Body mass index**		
BMI < 25	1.00	1.00
BMI ≥ 25	1.91 [0.94,3.89]	1.19 [0.66,2.17]
**At least moderate physical activity**		
No	1.22 [0.45,3.29]	2.1 [1.09,4.02]
Yes	1.00	1.00
**Family history of Diabetes Mellitus**		
No	1.00	1.00
Yes	2.9 [0.82,10.1]	7.07 [3.07,16.25]
**Waist circumference**		
Low risk	1.00	1.00
High risk	0.92 [0.21,4.13]	1.19 [0.65,2.17]
**Alcohol consumed the last 30 days**		
No	1.00	1.00
Yes	0.54 [0.16,1.81]	0.47 [0.15,1.47]

**Table 3:** Multivariate analysis of factors associated with diabetes mellitus among urban and rural residents of Gondar, North West Ethiopia (2012)

	**Adjusted OR [95% CI]**	
**Variable**	**Rural**	**Urban**
**Age in year**		
35-44	1.00	1.00
45-54	1.07 [0.39,2.98]	1.60 [0.62,4.17]
55-64	0.27 [0.03,2.13]	5.26 [2.21,12.5]
≥ 65	1.23 [0.36,4.17]	4.86 [1.99,11.9]
**Sex**		
Male	1.00	1.00
Female	1.07 [0.41,2.79]	1.20 [0.57,2.56]
**Body mass index**		
BMI < 25	1.00	1.00
BMI ≥ 25	0.64 [0.23,1.79]	1.19 [0.66,2.17]
**At least moderate physical activity**		
No	1.00	1.00
Yes	0.62 [0.15,2.65]	0.52 [0.29,0.94]
**Family history of Diabetes Mellitus**		
No	1.00	1.00
Yes	8.46 [0.61,117]	5.05 [2.43,10.51]
**Waist circumference**		
Low risk	1.00	1.00
High risk	1.45 [0.75,2.81]	1.04 [0.54,2.00]
**Alcohol consumed the last 30 days**		
Yes	1.00	1.00
No	4.16 [1.02,17.04]	1.44 [0.61,3.42]

^*^ Adjusted for scio-demogrpahic, life style and anthropometric measurements.

## Discussion

In this population-based cross-sectional study, we were able to measure the prevalence of diabetes among adults aged 35 years and older in rural and urban settings of northwest Ethiopia. We also uncovered that a large proportion of DM is undiagnosed and untreated in this population. The risk factors included family history of DM, older age and physical inactivity in the urban population and moderate alcohol drinking in the rural population.

A significant difference was observed in the prevalence of DM among urban and rural dwellers in the study area. The prevalence among the rural dwellers turned out to be very close to that of the rural dwellers in West Africa (2.6%) [20]. Even though the prevalence among urban dwellers of this study is higher than that of rural dwellers, it is comparatively lower than the prevalence seen among the Commercial Bank employees in Addis Ababa (6.5%) [5] and a similar study done on urban dwellers of South Africa (12.1%) [21]. Differences observed in diabetes prevalence estimates across studies might be due to the socio-demographic and life style differences in the localities studied. Literature shows that urbanization and economic development are increasing the prevalence of DM by about 40% [22,23].

The proportion of previously undiagnosed DM cases was alarmingly high in both urban and rural settings, although the rate is much higher in rural populations. A similar study in rural South Africa has also reported 84.8% of undiagnosed DM cases [10,24]. The high rate of these findings may reflect the low awareness of the public and primary health care providers about the disease [25].

In the current study, increasing age was an important risk factor for DM in urban areas which is consistent with other reports [26,27]. Since aging causes a progressive decline in the strength and endurance of musculature, which causes muscle atrophy, the risk of developing Type 2 DM increases.

Similarly, a study conducted in Puerta Rico showed that the prevalence of DM increased with age, with a peak prevalence in the urban oldest age-group [28]. The decrease in physical activity associated with city lifestyle partly explains the excess prevalence of diabetes mellitus in such groups. The variation could be explained by the differential distribution in risk factors between urban and rural dwellers across different age groups in populations. In rural settings, even older people are likely to be physically active compared to their urban counterparts. Moreover, our finding shows that a markedly high proportion of BMI was noted among older age urban dwellers. This exposure is likely to be responsible for the observed high rate of prevalence in this age group.

Studies show that individuals with a family history of diabetes are at increased risks for DM. In our findings, family history of DM was the major independent variable associated with DM in urban areas. This finding is consistent with other reports [29-31]. How genetic predisposition alone causes DM is not known, but life style and living environments within families are perhaps most likely to be the contributing factors [32].

Our finding is in agreement with studies from sub-Saharan Africa in indicating the beneficial effect of physical activity in reducing the risk of Type 2 DM [23,33,34]. Also, a systematic review of prospective cohort studies shows that lack of physical activity of moderate intensity has a risk of Type 2 diabetes [35]. The reduction in physical activity associated with a more sedentary lifestyle in urban areas may partly explain the development of diabetes mellitus in these areas. Exercise has been shown to increase insulin-stimulated glycogen synthesis and increased muscle mass which can contribute to the beneficial effects of physical activity on insulin sensitivity [36].

Even though quantification of the dose and duration of alcohol was not made in this study, alcohol consumption was inversely associated with high fasting blood glucose among the rural study population. About 81% of the rural study population reported regular alcohol consumption. Rural populations in the study area prepare local beer known as *tella*. The preparation has no standards and the alcohol content is highly variable. Previous research has indicated that low to moderate alcohol consumption is associated with a decreased incidence of diabetes mellitus compared with lifetime abstainers [33,37-39]. Further study on alcohol consumption patterns and the alcohol content of locally prepared alcoholic drinks is important to clearly understand the association with diabetes.

The limitation of this study is that it was only a cross-sectional design which might not show temporal relationships and thus the observed associations may not necessarily be causal. The inclusion of only adults above the age of 35 years could also affect the overall prevalence, and since the Ethiopian population has a very broad base, the likely effect is that the overall adult prevalence of diabetes is likely to be lower than we reported in this paper. We included the older age group to increase our chances of getting diabetic cases for the factor analysis due to the limitation in resources. Lack of some details on exposures such as alcohol consumption, and Khat chewing smoking is another limitation; although khat chewing and smoking are generally extremely low among adults of this age in the study area. Also some of the information was based on self-reporting which is subject to recall bias.

We were also not able to distinguish the various types of DM in this study. The sample size was not also sufficient to do rigorous and detailed analyses on the various risk factors. This study can be regarded an eye opener for studies on chronic diseases in rural population of Ethiopia and clearly indicates the need for further detailed research with sufficiently large sample sizes.

## Conclusion

The prevalence of diabetes mellitus is considerably higher among urban dwellers compared to the rural population. Overall, a large majority of diabetic cases in the study identified were not previously diagnosed and treated. The proportion of undiagnosed diabetes was higher among the rural population. Appropriate actions need to be taken to avail diagnostic and treatment services in all health units to detect DM early and initiate appropriate preventive and supportive care before complications arise. Physical activity among urban dwellers is recommended.

## Competing interests

The funders had no role in the study design, data collection, analysis, decision to publish, or in the preparation of the manuscript. The authors declare no competing interests.

## Authors’ contributions

SMA, YB, and AW conceived and designed the study. SMA analyzed the data and wrote the draft manuscript. AA commented on the manuscript. YB & AW commented on the draft and approved the final manuscript. All authors read and approved the final manuscript.

## Pre-publication history

The pre-publication history for this paper can be accessed here:

http://www.biomedcentral.com/1471-2458/14/97/prepub
